# The cGAS-STING pathway and female reproductive system diseases

**DOI:** 10.3389/fimmu.2024.1447719

**Published:** 2024-10-09

**Authors:** Ruijie Li, Hengwei Liu, Yi Liu

**Affiliations:** ^1^ Department of Obstetrics and Gynecology, Union Hospital, Tongji Medical College, Huazhong University of Science and Technology, Wuhan, China; ^2^ Department of Obstetrics and Gynecology, Zhongnan Hospital of Wuhan University, Wuhan, China

**Keywords:** cGAS-STING, female reproductive system, endometriosis (EM), adenomyosis (AM), ovarian cancer

## Abstract

The cGAS-STING pathway has become a crucial role in the detection of cytosolic DNA and the initiation of immune responses. The cGAS-STING pathway not only mediates protective immune defense against various DNA-containing pathogens but also detects tumor-derived DNA to generate intrinsic anti-tumor immunity. However, abnormal activation of the cGAS-STING pathway by self-DNA can also lead to autoimmune diseases and inflammatory disorders. This article reviews the mechanisms and functions of the cGAS-STING pathway, as well as the latest research progress in female reproductive-related diseases. We focus on the regulatory mechanisms and roles of this pathway in common female reproductive disorders, discuss the clinical potential of the cGAS-STING pathway as biomarkers and therapeutic agents for female reproductive diseases, as well as the research controversies, technical issues, and biological knowledge gaps that need to be resolved. Furthermore, we provide new ideas for the treatment and prevention of these diseases.

## 1 Introduction

Innate immunity is the first line of defense against invading pathogens. Detection of foreign DNA is a key element of immunity in many organisms. In mammalian cells, this task is accomplished to a large extent by the cyclic GMP-AMP synthase (cGAS) stimulator of the interferon genes (STING) pathway (1, 2).

The cGAS-STING signaling pathway is an important pivot for cytosolic DNA sensation and cytokine induction. Typically, there are two main downstream routes of the cGAS-STING pathway to induce the production of cytokines such as interferon (IFN), one is the STING–TBK-1–IRF3 axis and the other STING–IKK–NF-κB axis.

The specific process is that when abnormal DNA is present in the cytoplasmic matrix, this kind of cytosolic DNA binds to and activates cGAS, which catalyzes the synthesis of 2 ‘ 3 ′ - cGAMP from ATP and GTP. 2 ‘ 3 ′ - cGAMP binds to the endoplasmic reticulum (ER) adaptor STING, which traffics to the ER - Golgi intermediate compartment (ERGIC) and the Golgi apparatus. STING then activates TBK-1 and IKK. TBK1 phosphorylates STING, which in turn recruits IRF-3 to be phosphorylated by TBK-1 (3, 4). Phosphorylated IRF-3 dimerizes and then enters the nucleus, inducing the expression of type I interferon (IFN1) and other inflammatory cytokines such as TNF, IL-1b, and IL-6 (5, 6). STING also releases NF-kB into the nucleus through activation of the kinase IKK (7), which acts synergistically with IRF3 to amplify the induction of IFN, as well as other immunomodulatory molecules (8).

In eukaryotic cells, DNA is present in the nucleus and mitochondria, and the cytoplasmic matrix is free of DNA. However, DNA-containing microorganisms can deliver DNA into the cytoplasm by infecting the host cells, and the cytosolic DNA activates the cGAS-STING pathway to open up the protective immune defense of the body. In addition, when tumor cells are ingested by phagocytes, tumor DNA may escape the phagosomes and enter the cytoplasm to activate cGAS and its downstream molecules (6). The detection and innate perception of tumor-derived DNA can promote anti-tumor immunity. Although in general, the immune system does not recognize an organism’s DNA, the cGAS-STING pathway will be activated when nuclear DNA or mitochondrial DNA leaks pathologically into the cytosol (9, 10) or gain-of-function mutations in STING lead to constitutive STING activation (11), resulting in an excessive autoimmune response.

The cGAS-STING pathway is involved in the regulation of multiple diseases due to its important role in infection, immunity, and inflammation. In recent years, more and more studies have shown that the abnormal activation or inhibition of the cGAS-STING signaling pathway is closely related to the occurrence and development of a variety of female reproductive system diseases. Some studies have found that the cGAS-STING pathway is over-activated in certain female reproductive system diseases, leading to an increased inflammatory response that promotes the development of the disease. Moreover, the cGAS-STING pathway is involved in regulating the immune balance in the female reproductive system, and the aberrant activation of this pathway may lead to an attack by the immune system on normal tissues, which can cause autoimmune diseases. In some tumor types, tumor-associated immune escape mechanisms inhibit the activation of the cGAS-STING pathway. There are also new findings suggesting that the cGAS/STING-dependent signaling networks affect female reproductive disorders by modulating autophagic degradation or various modes of cell death (e.g., apoptosis, necrosis, iron death, mitotic cell death, and immunogenic cell death). Although studies on the relationship between the cGAS-STING pathway and female reproductive system diseases are still relatively limited, the research in this field is deepening.

In this review, we highlight the mechanism and function of cGAS-STING pathway and in how cGAS-STING pathway works in female reproductive–related diseases. We also discuss the clinical potential of cGAS-STING pathway as biomarkers and therapeutic agents in female reproductive diseases as well as research controversies, technical issues, and biological knowledge gaps that need to be addressed. Here we review the recent advances in understanding the cGAS–STING pathway, focusing on the regulatory mechanisms and roles of this pathway in common female reproductive disorders, and provide new ideas for the treatment and prevention of these diseases.

## 2 Overview of the cGAS-STING signaling pathway

### 2.1 The discovery of cGAS-STING signaling pathway

The cellular innate immune system is critical for recognizing pathogenic infections and establishing effective host defenses. However, the key molecular determinants that promote appropriate immune responses following DNA virus infections remain to be identified. In 2008, Barber et al. first identified and characterized STING (Stimulator of Interferon Genes) proteins through two independent cDNA overexpression screens and confirmed their key role in the natural immune response (12). They found that STING consists of five transmembrane regions, predominantly resides in the endoplasmic reticulum, and is able to activate the NF-κB and IRF3 transcriptional pathways to induce the expression of type I interferons and exert potent anti-viral effects. In contrast, the absence of STING rendered murine embryonic fibroblasts highly susceptible to infection by negative-stranded viruses, including vesicular stomatitis virus. During the same period, it was proposed that STING (then known as MPYS) was associated with major histocompatibility complex class II (MHC II) and involved in pro-apoptotic signaling (13).

Related studies indicate that STING has TBK1/IRF3-dependent antiviral activity, but the upstream signals by which it exerts it signaling effects are unknown. Although STING is commonly thought to be involved in double-stranded DNA recognition (12, 14), more pronounced phenotypes have indeed been observed in DNA viruses or double-stranded DNA ligands. The solution to this conundrum comes from the pioneering work of Chen et al. In 2013, Chen and his colleagues (15) showed that mammalian cytoplasmic extracts synthesize cyclic guanosine monophosphate-adenosine monophosphate (cyclic GMP-AMP or cGAMP) from adenosine triphosphate and guanosine triphosphate *in vitro* in the presence of DNA but not RNA. DNA transfection or DNA viral infections in mammalian cells also trigger the production of cGAMP. cGAMP subsequently binds to STING, leading to the activation of IRF3 and inducing the production of IFN-β. cGAMP thus acts as an endogenous second messenger and triggers the production of interferon in response to cytoplasmic DNA. After identifying cGAMP, they focused on cytoplasmic extracts with cGAMP synthetic activity and identified cGAMP synthase (cGAS) by biochemical purification and quantitative mass spectrometry. cGAS is a cytosolic DNA sensor that catalyzes the production of the second messenger, cGAMP, by binding to DNA in the cytoplasm to induce interferon (16). The subsequent study (17) has shown that endogenous cGAMP in mammalian cells contains two different phosphodiester linkages, one between 2’-OH of GMP and 5’-phosphate of AMP, and the other between 3’-OH of AMP and 5’-phosphate of GMP. Among them, the binding affinity of 2’3’-cGAMP to the adaptor protein STING was much higher than that of cGAMP molecules containing other phosphodiester linkage combinations.

The discovery of cGAS and cGAMP greatly compensates for the lack of dsDNA recognition and further refines the way the cGAS-STING pathway signals in the cell. It is beneficial for researchers to conduct more in-depth studies on its role in sensing pathogen infection, tumor immunity, and autoinflammatory.

### 2.2 Basic structural features of cGAS and STING

#### 2.2.1 cGAS structure

Cyclic GMP-AMP synthase (cGAS) comprises a disordered N-terminal domain and a C-terminal catalytic domain. The C-terminal catalytic part harboring the nucleotidyltransferase domain contains positively charged DNA-binding sites, namely a primary site (site A) and a secondary site (site B). The binding of DNA to the A site triggers the activation of cGAS mainly by causing conformational adjustments around the catalytic site, facilitating an optimal interaction with the ATP and GTP substrates. The adjacent B-site also interacts with DNA, thus influencing cGAS activation (2, 18). To achieve a stable active conformation, cGAS needs to form a dimer, with two DNA strands positioned between the two cGAS protomers (19, 20). Each of the two DNA molecules bound by the dimer is attached to site A of one cGAS protomer and to site B of the corresponding other protomer. The 2:2 complex plays a crucial role in cGAS-mediated signaling, as mutations of key residues in the secondary DNA-binding site of cGAS or disruptions to the cGAS dimerization interface impair the enzymatic activity of cGAS.

While DNA ligands shorter than 20 bp are capable of binding cGAS and supporting the formation of the 2:2 complex, complete activation of cGAS catalytic activity *in vitro* and *in vivo* signaling necessitates longer DNA. On long double-stranded DNA molecules, multiple cGAS dimers arrange themselves into ladder-like assembly (21). The 2:2 cGAS-DNA complex formation prearranges the two DNA molecules in a nearly parallel orientation, enhancing their ability to bind subsequent cGAS dimers. This results in a high level of cooperativity in cGAS recruitment and activation. This mode of activation provides a vital built-in safeguard mechanism for living cells, ensuring that cGAS signaling is initiated only when longer stretches of double-stranded DNA surpass a specific signaling threshold. In contrast, spurious activation of cGAS on limited and short double-stranded DNA is averted.

#### 2.2.2 STING structure

STING is a transmembrane protein located in the endoplasmic reticulum (ER) that contains a short N-terminal cytosolic segment, four transmembrane (TM) helices that localize to the ER membrane, a cytosolic ligand-binding domain (LBD), and a C-terminal tail (CTT) that is responsible for binding TBK1. The STING exists as a constitutive hydrophobic dimer with a double-wing butterfly-like structure. The two LBDs create a ligand-binding pocket in a deep V-shaped cleft between the dimer subunits, serving as the functional unit capable of binding 2’3’-cGAMP (22–25). All CDNs adopt an overall U-shape structure and bind in similar modes to the central crevice of the deep V-shaped STING dimer. The phosphate and ribose moieties are positioned at the bottom of the crevice, whereas the two bases are situated above and oriented roughly parallel to each other. The LBD firmly embraces the ligands through numerous contacts, which is the basis for the high affinities and specificities of the interactions.

STING undergoes extensive conformational rearrangements upon binding to 2’3’-cGAMP, in which the two wings of the LBD rotate inward toward each other and form an ordered β-sheet, leading to the closure of the ligand-binding pocket. The LBD of STING is 180° opposite its TM domain and exposes a CTT. This conformational change results in the release of STING from the ER, which is eventually translocated to the ER-Golgi intermediate compartment (ERGIC) through coat protein complex II (COP-II) vesicles (23, 26). The rotation of the LBD assists in the arrangement of the STING dimer in a parallel fashion and the formation of the STING tetramer and higher-order oligomers through side-by-side packing (27). Oligomerization is further promoted by disulfide bridges that extend across separate STING dimers as well as by palmitoylation of cysteine residues, C88 and C91 (11, 28). High-order oligomerization of STING creates a signaling platform that recruits and activates TBK1. This results in the more efficient phosphorylation of the higher-order STING oligomers compared to the dimers (29).

### 2.3 Molecular mechanism of cGAS-STING signaling

cGAS is a cytosolic DNA sensor, which contains a nucleotidyltransferase domain and two major DNA binding domains. In the absence of DNA, cGAS exists in an autoinhibited state (2, 19, 20). When abnormal DNA is present in the cytoplasm, cGAS is activated by these dsDNAs. These dsDNAs can be derived not only from non-self DNA (e.g., viral DNA, intracellular bacterial and protozoan DNA, engulfed tumor-derived DNA) but also from extracellular self DNA (e.g., DNA translocated to the cytosol via exosomes and microvesicles) and intracellular self DNA (e.g., mitochondrial and nuclear DNA). cGAS binds to DNA to form a 2:2 complex after recognizing it, and binding to DNA induces a conformational change in the active site, which catalyzes the synthesis of 2’3’-cGAMP from ATP and GTP (15, 16, 19, 20).

2’3’-cGAMP is able to activate STING proteins located on the endoplasmic reticulum membrane and binds to the V-shaped structure of the STING dimer, resulting in a conformational change of STING. This conformational change leads to the release of STING from the ER, which penetrates into COP2 vesicles for translocation to the ER-Golgi intermediate compartment (3, 30). During this process, the carboxyl terminus of STING recruits and activates the kinase TBK1.The recruited TBK1 undergoes autophosphorylation, phosphorylating both STING and IRF3.STING phosphorylated by TBK1 has an increased affinity for the interferon regulatory factor IRF3, which promotes the recruitment of IRF3. Phosphorylated IRF3 dimerizes and translocates to the nucleus and induces type I IFN expression and secretion (4, 31, 32).

**Figure 1:**
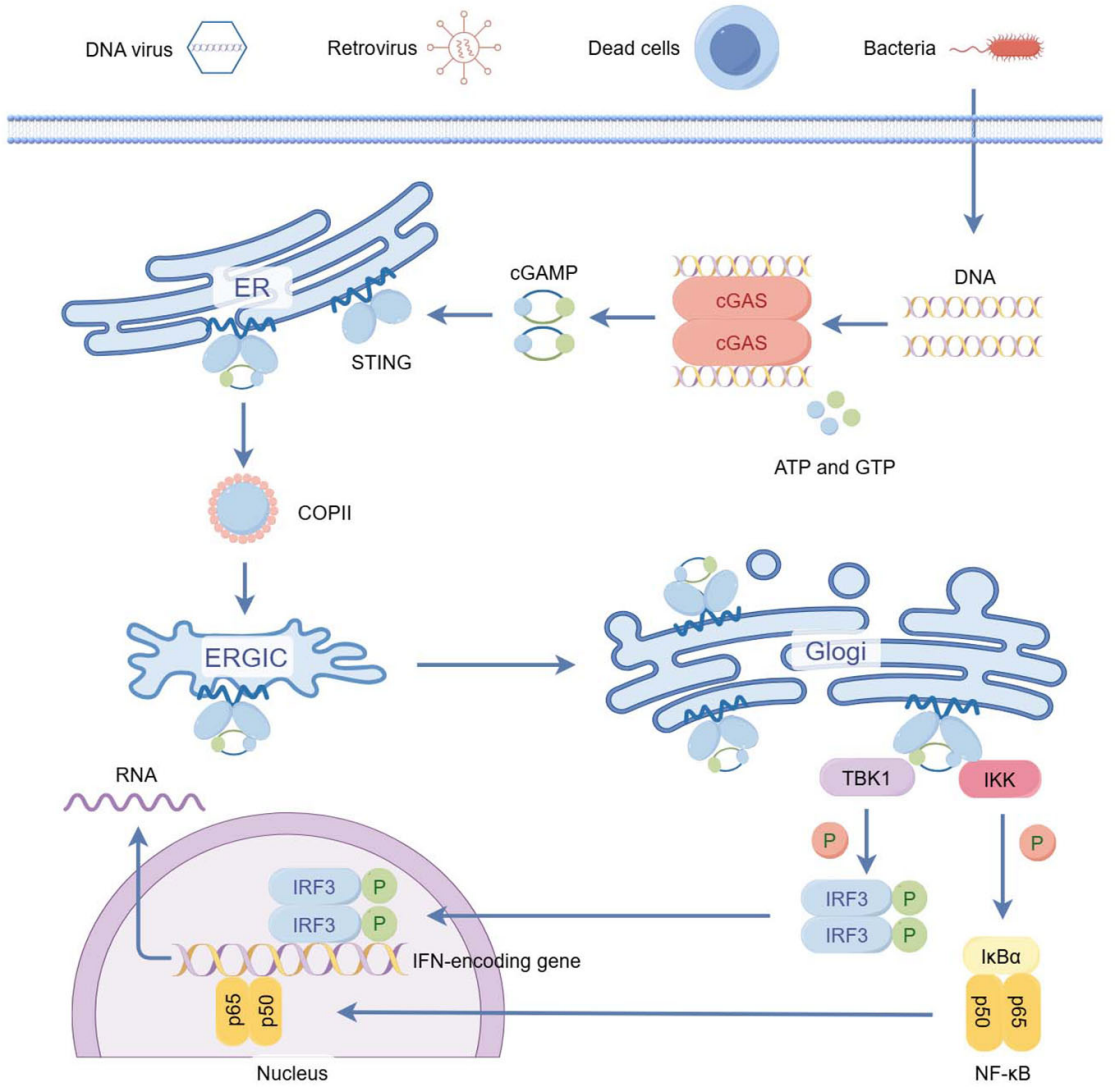
The cGAS–STING pathway of cytosolic DNA sensing. (By Figdraw) cGAS-STING is part of the innate immune system activated by cytosolic DNA. The specific process is that when abnormal DNA is present in the cytoplasmic matrix, this kind of cytosolic DNA binds to and activates cGAS, which catalyzes the synthesis of 2 ‘ 3 ′ - cGAMP from ATP and GTP. 2 ‘ 3 ′ - cGAMP binds to the endoplasmic reticulum (ER) adaptor STING, which traffics to the ER - Golgi intermediate compartment (ERGIC) and the Golgi apparatus. STING then activates TBK-1 and IKK. TBK1 phosphorylates STING, which in turn recruits IRF-3 to be phosphorylated by TBK-1. Phosphorylated IRF-3 dimerizes and then enters the nucleus, inducing the expression of type I interferon (IFN1) and other inflammatory cytokines such as TNF, IL-1b, and IL-6. STING also releases NF-kB into the nucleus through activation of the kinase IKK, which acts synergistically with IRF3 to amplify the induction of IFN, as well as other immunomodulatory molecules.

STING also activates IκB kinase (IKK), which phosphorylates the IkB family of inhibitors of the transcription factor NF-kB. The phosphorylated Ik B protein is degraded via the ubiquitin-proteasome pathway, releasing NF-kB into the nucleus. NF-κB, along with other transcription factors, acts synergistically with IRF3 by collectively binding a specific enhancer region (called the enhanceosome) within the IFN promoter, inducing abundant expression of interferons and inflammatory cytokines such as TNF, IL-1b, and IL-6, and ultimately triggers the corresponding immune response (12, 33) (**Figure 1**).

## 3 Function role of the cGAS-STING signaling pathway in female reproductive diseases

### 3.1 cGAS - STING and endometriosis

Endometriosis (EM) is a prevalent gynecological condition characterized by the presence of endometrial tissue outside the uterine cavity. EM significantly impacts the quality of life for many women, often leading to infertility and chronic pelvic discomfort. The underlying mechanisms of EM involve the migration, invasion, and angiogenesis of endometrial tissue outside the uterus (34). The retrograde menstruation hypothesis, which suggests that endometrial fragments enter the pelvic cavity during menstruation, implant onto the peritoneum and abdominal organs, and cause inflammation and adhesion formation, is a widely accepted explanation for EM. However, it doesn’t account for why only 10% of women with retrograde menstruation develop EM despite its occurrence in 90% of women (35, 36).

Recent research has shed light on the role of inflammation, oxidative stress, and angiogenesis in EM’s pathogenesis (37–39). The cGAS-STING signaling pathway, known for initiating innate host immunity in response to cytoplasmic DNA, is under investigation for its potential role in EM. Previous studies indicated that abnormal activation of cGAS-STING signaling pathway was tightly associated with the local inflammation and immune dysregulation in EM.

Zhu et al. found elevated expression of cGAS-STING signaling and autophagy levels in both epithelial and stromal tissues of ectopic endometrium. They also discovered that the cGAS-STING signaling pathway upregulates autophagy. Moreover, STING antagonists effectively reduced both autophagy levels and the size of endometriosis lesions (40). Autophagy upregulation is known to promote the migration and invasion of human endometrial stromal cells (HESCs) (41). Therefore, the overexpression of the cGAS-STING signaling pathway enhances the migration and invasion capacity of HESCs by activation of autophagy. Moreover, Qu et al. demonstrated that STING expression was higher in the epithelium of ectopic endometrium, correlating with intraepithelial lymphocyte infiltration. This finding suggested a role for STING in promoting chronic inflammation in ectopic endometrium. However, no significant difference in STING expression was observed in endometrial stromal cells between ectopic and eutopic endometrium (42). While there is a dispute about whether STING expression is elevated in ectopic endometrial stromal cells, it is certain that STING expression is elevated in the ectopic endometrial epithelium. This overexpression contributes to the development of ectopic endometrial foci.

The understanding of how the cGAS-STING signaling pathway influences endometriosis requires further exploration. First and foremost, the precise origin of the DNA that activates this pathway remains unidentified. While cryptic viral or bacterial infections are potential triggers, direct evidence is currently lacking. Another conjecture is that the self-DNA triggers an autoimmune attack, which is associated with the pathological leakage of nuclear DNA or mitochondrial DNA into the cytoplasm (9, 10). For example, exposure to cellular stress or inflammasome agonists can trigger mitochondrial damage and enhance the production of mitochondrial reactive oxygen species (mROS), resulting in the release of oxidized mtDNA (OX-mtDNA) into the cytosol to activate the cGAS-STING pathway (43). Secondly, specific STING variants capable of constitutively inducing a type I interferon response could also play a significant role (11). For instance, patients with SAVI (STING-associated vasculopathy with onset in infancy) have a point mutation in exon 5 of STING (V147L, N154S, or V155M), which exhibit a gain-of-function phenotype and are capable of stimulating the production of an IFNβ reporter construct that causes early-onset systemic inflammation (44, 45). Thirdly, the cGAS-STING pathway has been found to influence several other diseases’ progression by regulating apoptosis (46), glycolysis (47) and iron death (48), etc. Given these observations, it is reasonable to speculate that the cGAS-STING pathway could also impact endometriosis through diverse mechanisms.

### 3.2 cGAS - STING and adenomyosis

Adenomyosis(AM) stands as a prevalent gynecological disorder, characterized by the histological presence of endometrial glands and stroma within the myometrium (49). Common symptoms are dysmenorrhoea, menorrhagia, and infertility, although many women have no symptoms (50, 51). Several theories have been proposed for the etiology of adenomyosis, including damage to the endometrial basal layer (11, 29) and local estrogen excess, which may facilitate the downward endometrial growth into the myometrium (4, 30, 31). However, the pathophysiology of adenomyosis remains poorly understood (4, 32). Recently, there has been increasing evidence that abnormal immune responses may also play an important role in the pathogenesis of adenomyosis (52–54).

The cGAS-STING pathway as a popular molecular pathway in the regulation of the immune system has been shown to be involved in the pathogenesis of adenomyosis. Lin et al. found that, compared with control patients without adenomyosis, the lesional tissues of patients with adenomyosis showed significantly increased expression of key cGAS-STING pathway factors including cGAS, STING, TBK-1, IFN-α, IFN-β, and TNF-α. And this expression pattern was not related to the type of adenomyosis, with both diffuse and focal adenomyosis showing an increase of cGAS-STING signals. It was also found that the main pathway for IFN1 production in adenomyosis was the STING–TBK-1–IRF3 axis, rather than the STING–IKK–NF-κB axis (55).

High expression of cGAS-STING and their downstream factors in adenomyotic foci may continuously activate the inflammatory response and maintain the inflammatory microenvironment in the lesions. These effects may lead to tissue damage and abnormal growth, cause abnormal angiogenesis, disrupt the normal boundary between the endometrium and myometrium, and facilitate the invasion of endometrial epithelial and/or stromal cells into the myometrium via epithelial-mesenchymal transition pathways (35). Thus, the inhibition of the key target gene of the cGAS-STING pathway may contribute to the treatment of adenomyosis.

The mechanism of cGAS activation in adenomyosis requires further investigation. The expression of apoptotic genes has been observed in adenomyosis foci (56, 57). Apoptotic or necrotic cells may abnormally release nuclear DNA or mitochondrial DNA for cGAS recognition. This may be the subject of future research. Additionally, recent research by K. Wang et al. has shown that hypoxia induces an increase in the proliferation and migration capabilities of endometrial stromal cells (ESC), accompanied by the activation of the cGAS-STING signaling pathway and elevated IFN-α levels, promoting the development of AM (58). AM lesions undergo repeated tissue injury and repair, accompanied by increased endometrial fibrosis, leading to hypoxia (59). Hypoxia can trigger excessive activation of mitochondrial autophagy, leading to mitochondrial structural and functional impairments and the release of mtDNA into the cytoplasm (60). This process can activate the cGAS-STING signaling pathway, thereby enhancing type I interferon-mediated autoimmune responses to promote the development of a chronic pro-inflammatory state (61).

### 3.3 cGAS - STING and ovarian cancer

Ovarian cancer is the fifth most common cancer in women and the deadliest of the gynecological malignancies (62–64). The majority of ovarian tumors originate in the epithelium. Direct spread, intraperitoneal implantation, and lymphatic metastasis are the main metastatic routes for ovarian malignancies. Despite advances in cytotoxic therapies, only 30% of patients with advanced-stage ovarian cancer survive 5 years after initial diagnosis. Therefore, an understanding of the molecular mechanisms that regulate the escape of ovarian cancer cells from immune surveillance has the potential to have a significant impact on the outcomes of this devastating disease (65, 66).

Interferon (IFN) has a crucial role in the immune surveillance of cancer cells. In addition to its direct cytotoxic effect on cancer cells, IFN promotes the recruitment of anti-tumor immune cells including T cells, B cells, natural killer (NK) cells, macrophages, and dendritic cells (DCs), which act as intrinsic barriers to anti-tumorigenesis (8). CGAS-STING can cause the production of immunostimulatory factors such as IFN and activate a variety of immune cells, thus playing an important role in anti-tumor immunity (6, 67).

In response to the tumor-suppressor function of cGAS-STING, cancer cells tend to downregulate these proteins (68). Loss-of-function mutations or epigenetic silencing of the cGAS/STING promoter regions have been observed in a variety of tumors. In addition, some missense STING variants are unable to activate cytokine production following exposure to cytokine DNA or DNA-damage events, thus allowing cancer cells to avoid anti-tumor immune responses (69).

In ovarian cancer, it has been found that the copy number of the deubiquitinating enzyme USP35 is higher in cancer tissues than in normal ovarian or blood samples and that cancer cells directly deubiquitinate and inactivate STING by upregulating USP35 (70). Another potential factor is SETDB1. SETDB1 is a molecule that is frequently over-expressed in human cancers (71). Micronuclei are cytosolic DNA generated by unrepaired DNA lesions and/or mitotic defects during the G_2_-M-phase of the cell cycle (72). SETDB1–TRIM28 inhibition led to micronuclei formation in the cytoplasm, which is known to activate the cGAS–STING pathway. Overexpression of SETDB1-TRIM28 was found in several ovarian cancer cell lines, thereby inactivating the cGAS-STING pathway (73).

Whereas, further studies are still needed to clarify the expression levels of cGAS/STING in ovarian cancer tissues. The study by de Queiroz et al. found that cGAS and STING expression levels were decreased in most ovarian cancer tissues compared to normal ovarian tissues, and the loss of cGAS/STING was more obvious with higher tumor grade and severity (74). However, Jutta Huvila et al. found that STING expression differs among ovarian cancer histotypes; low-grade serous ovarian carcinomas and serous borderline tumors have uniform high STING expression, while high-grade serous and endometrioid carcinomas have heterogeneous expression, and clear cell and mucinous carcinomas show low expression. High STING expression may reflect pathway activation or histogenesis and the mechanisms may be different in different ovarian carcinoma histotypes (75). The molecular drivers of these phenomena are not fully understood. Overall, STING expression appears to have different roles in different ovarian cancer histotypes and different contexts. Thus, it is worth considering whether therapeutic interventions that trigger innate immune activation are applicable to all subtypes of ovarian cancer. Future functional experiments are needed to elucidate the role of STING in ovarian carcinomas and to assess the integrity of the cGAS-STING pathway, which will help to provide more appropriate therapeutic strategies for different ovarian cancer subtypes.

### 3.4 cGAS - STING and cervical cancer

Cervical cancer is the most common gynecological malignant tumor, with a high incidence at the age of 50-55 years. The main metastatic modes are direct spread and lymphatic metastasis, and hematogenous metastasis is relatively rare. HPV infection is a high-risk factor for cervical cancer, with more than 80% of cervical cancers caused by infection with high-risk subtypes of HPV (HPV-18 and HPV-16) (76, 77).

HPV infection usually triggers the body’s immune response, which in most cases eliminates the infected cells. However, on the other hand, HPV has mechanisms to evade the body’s immune system, which can lead to the persistence of HPV infection and the possible progression to cervical cancer. For example, HPV16 activates a unique NF-κBp50-p65 and ERα inhibitory complex that suppresses the transcription and function of the double-stranded DNA innate sensor TLR9. This event leads to an inhibition of IFN production (78). HPV oncoproteins E6 and E7 can inhibit the IFN-1 pathway by suppressing activation of Signal Transducer and Activator of Transcription 1 (STAT1) (79). In addition, HPV oncoproteins E5 and E7 inhibit the ability of host cells to present HPV antigens by downregulating human leukocyte antigen I(HLA-I) expression (80–82).

Recent research has revealed a strong link between the cGAS-STING pathway and HPV infection. Notably, cGAS/STING expression is significantly lower in HPV-positive cervical cancer cells compared to HPV-negative cells. This phenomenon may be attributed to the interaction of HPV18 E7 protein with STING, which disrupts STING signaling (83). Furthermore, E7 selectively inhibits STING-mediated NF-κB activation, leading to reduced production of inflammatory cytokines (84). Similarly, E2 proteins encoded by high-risk HPV strains can downregulate the transcription of STING and IFN-κB, along with their downstream target genes. This downregulation likely represents an immune evasion mechanism employed by HPV to promote persistence and contribute to cervical cancer development (85).

In addition to the HPV virus reducing the expression of the cGAS-STING signaling pathway, polymorphisms within the cGAS gene itself can also influence susceptibility to cervical precancerous lesions. rs311678 is located in the intron 3-4 region of the cGAS gene. G allele in cGAS rs311678 might reduce the risk of cervical precancerous lesions and have antagonistic effects with HPV infection (86). This phenomenon is likely due to the rs311678 variant leading to increased levels of cGAS transcripts and, consequently, higher cGAS-STING pathway activity, which may interfere with the process whereby HPV encodes antagonists to evade innate immunity detection of HPV viral DNA (83, 87). Furthermore, studies have shown that mouse cGAS possesses structures that bind to 18bp double-stranded DNA (dsDNA) through two distinct binding sites, forming a 2:2 complex. Researchers hypothesize that the G genotype of rs311678 might influence these binding sites, potentially increasing their affinity for HPV DNA. This stronger affinity could lead to more efficient activation of the cGAS-STING pathway, ultimately resulting in a decreased risk of cervical precancerous lesions (19, 88).

In STING-deficient cervical cancer cells, the degree of STING absence was positively correlated with tumor growth. STING agonists are being increasingly developed as new agents for the treatment of cervical cancer.

#### 3.5 cGAS - STING and endometrial cancer

Endometrial cancer (EC) is a group of epithelial malignant tumors occurring in the endometrium, the incidence of this disease is increasing year by year and has become a major threat to women’s health. The typical age-incidence curve for endometrial cancer shows that most cases are diagnosed after menopause, with the highest incidence around the seventh decade of life (89). The vast majority of endometrial cancers are thought to be associated with sex hormone dependence. Due to the increased expression of local estrogen receptor (ER), the endometrium undergoes hyperplasia, atypical hyperplasia, and then carcinoma under long-term estrogen exposure (90). Recent studies bolster the hypothesis that immune escape mechanisms are the driving force behind EC progression. This hypothesis is supported by the abnormal expression of multiple inflammatory cytokines (e.g. IL-6, IL-8, TGF-β, VEGF, and IL-1Ra) in EC tissues (91). Furthermore, various immune cell types, including macrophages, natural killer cells, and T helper cells, have been implicated in EC development (92). Intriguingly, the cGAS-STING pathway has been shown to play a crucial role in both the production of these inflammatory cytokines and the activation of immune cells’ anti-tumor response.

**Figure 2:**
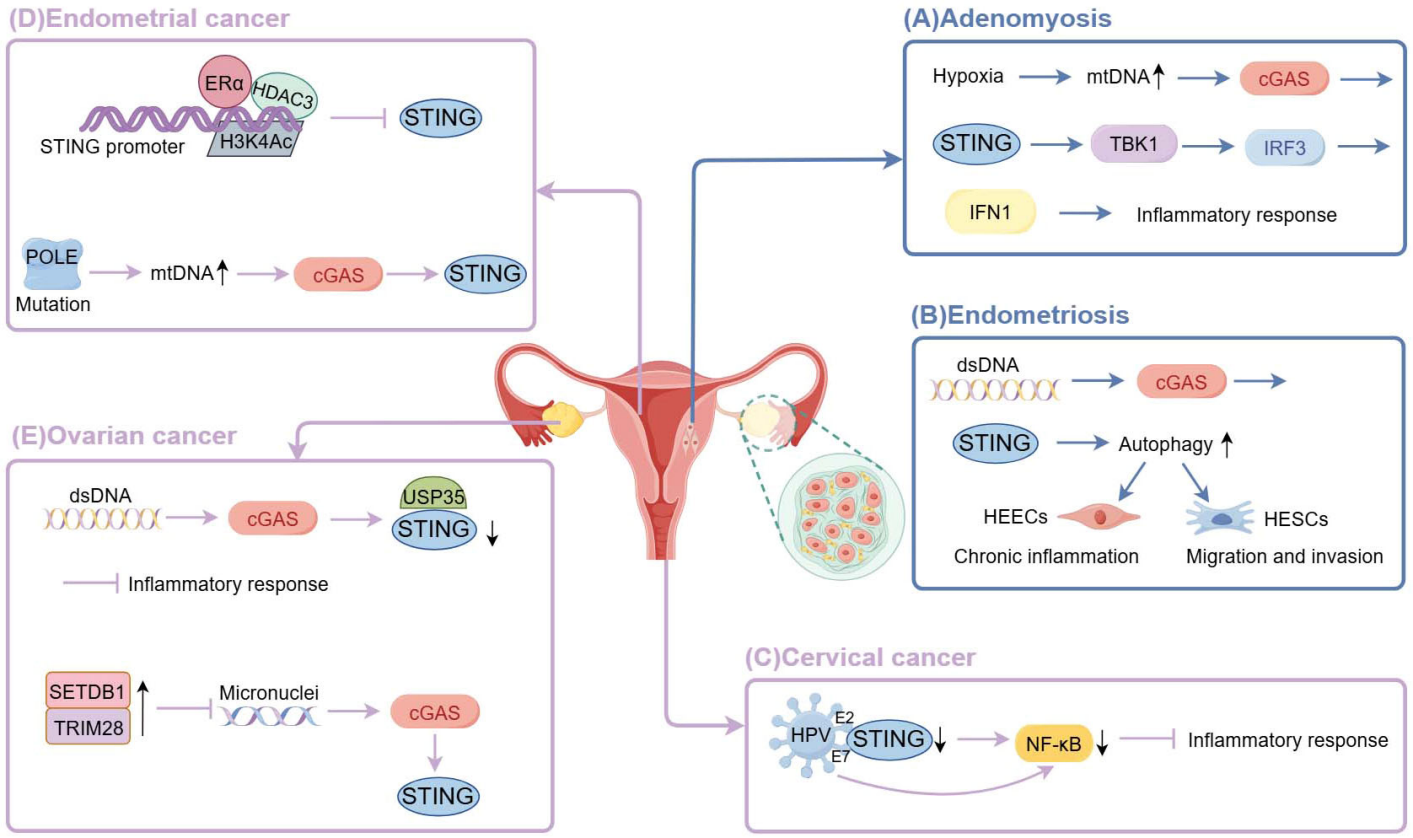
The cGAS-STING signaling in female reproductive system diseases. (By Figdraw) **(A)** Hypoxia of adenomyosis lesions triggers overactivation of mitochondrial autophagy, leading to mitochondrial structural and functional dysfunction and the release of mtDNA into the cytoplasm, thereby activating the cGAS-STING signaling pathway and producing IFN1 through the STING-TBK-1-IRF3 axis to persistently activate the inflammatory response. **(B)** Cytosolic DNA promotes cGAS-STING signaling to enhance the migration and invasion capacity of HESCs by upregulating autophagy, thus promoting the growth of ectopic endometrium; and to promote chronic inflammation in the ectopic endometrium through high expression in HEECs. **(C)** HPV E7 proteins bind to STING to block its signaling and selectively inhibit STING-triggered NF-κB activation; HPV E2 proteins reduce STING and IFN-κ transcription and their downstream target genes. **(D)** β-estradiol-ERα recruited HDAC3 and induced the deacetylation of histone 3 lysine 4 by binding to the STING promoter, thereby reducing the expression of STING.POLE mutation leads to genomic instability and increased DNA damage, upregulates cytoplasmic DNA, triggers cGAS, and initiates downstream signaling. **(E)** Cancer cells deubiquitinate and inactivate STING by upregulating deubiquitinating enzyme USP35. Overexpression of SETDB1-TRIM28 leads to reduced micronuclei formation, thereby inactivating the cGAS-STING pathway.

G. Chen et al. found that the expression of STING was largely reduced in endometrial cancer, and the expression of STING was regulated by β-estradiol and histone deacetylase3 (HDAC3). Mechanistically, β-estradiol-ERα recruited HDAC3 and induced the deacetylation of histone 3 lysine 4 by binding to the STING promoter, thereby reducing the expression of STING. And β-estradiol promoted ERα and HDAC3 to localize in STING promoters (93). Thus, inhibition of HDAC3 increases STING expression, thereby preventing cancer cell proliferation and inducing apoptosis, and effectively controlling type I and type II EC (94). The combination of HDAC inhibitors with chemotherapy has shown promising results in clinical trials (95). On the contrary, the P286R mutation in exon 9 of the DNA polymeraseϵ(POLE) gene activates intrinsic immunity in cancer cells and suppresses the occurrence of endometrial tumors via the cGAS-STING pathway (96). Specifically, the POLE mutation leads to genomic instability and increased DNA damage, upregulates cytoplasmic DNA, triggers cGAS, and initiates downstream signaling, thereby stimulating the expression of inflammatory genes and anti-tumor immune responses. Therefore, activating the cGAS-STING pathway through POLE mutations may help improve the survival of patients with endometrial cancer, and the stimulation of intrinsic immunity in cancer cells by POLE mutations provides a theoretical basis for personalized treatment of malignant tumors (**Figure 2**).

## 4 Drug development targeting the cGAS–STING signaling pathway

The cGAS-STING pathway is involved in a wide range of female reproductive disorders and has a significant impact on disease progression, which heralds its great potential as a drug target. For excessive inflammatory responses triggered by over-activation of the cGAS-STING pathway, cGAS or STING inhibitors can be considered. In contrast, for the treatment of malignant tumors, STING agonists may be used to enhance the body’s anti-tumor immunity, agonists targeting cGAS are rare. Currently, multiple preclinical studies involving drug-like compounds that selectively target cGAS or STING have shown promising results, which establish a solid foundation for ongoing clinical trials (**Table 1**).

**Table 1:** Inhibitors/agonists of cGAS-STING pathway in clinical trials.

Agent	Mechanism	First posted data	Conditions	Status	Phase	NCT Number
Hydroxychloroquine	cGAS inhibitors	2023/4/5	Systemic Lupus Erythematosus	Not yet recruiting	3	NCT05799378
Suramin	cGAS inhibitors	2020/8/3	Acute Kidney Injury	Active, not recruiting	2	NCT04496596
NO2-OA	STING inhibitors	2018/2/5	Primary Focal Segmental Glomerulosclerosis	Completed	2	NCT03422510
NO2- CLA	STING inhibitors	2015/5/5	Asthma	Completed	2	NCT02433977
ADU-S100	STING agonists	2019/5/3	Metastatic Head and Neck Cancer; Recurrent Head and Neck Cancer	Terminated	2	NCT03937141
DMXAA	STING agonists	2011/1/28	Advanced or Recurrent Solid Tumors	Completed	1	NCT01285453
MSA-2	STING agonists	2015/1/5	Infertility	Recruiting	4	NCT02330757
MK-1454	STING agonists	2020/1/7	Head and Neck Squamous Cell Carcinoma	Completed	2	NCT04220866
MK-2118	STING agonists	2017/8/15	Solid Tumor; Lymphoma	Completed	1	NCT03249792
BMS-986301	STING agonists	2019/5/21	Advanced Solid Cancers	Active, not recruiting	1	NCT03956680
GSK3745417	STING agonists	2019/2/18	Neoplasms	Active, not recruiting	1	NCT03843359
SB-11285	STING agonists	2019/9/20	Melanoma; Head and Neck Squamous Cell Carcinoma; Solid Tumor	Recruiting	1	NCT04096638
IMSA-101	STING agonists	2019/7/15	Solid Tumor	Completed	2	NCT04020185

### 4.1 cGAS inhibitors

The cGAS inhibitors can be broadly categorized into two main classes. The first class targets the cGAS catalytic site, competing with ATP or GTP substrates or the cGAMP product for binding. The second class competes with DNA for binding to cGAS, thereby impeding its initial activation.

PF-06928125 (97) exemplifies a compound that binds to the cGAS catalytic site, a region typically occupied by the adenosine base of ATP or cGAMP. Similarly, RU.521 (98) also selectively inhibits cGAS in a manner that occupies the active site of cGAS. RU.521 reduces the expression level of Ifnb1 mRNA in bone marrow-derived macrophages (BMDMs) from Trex1-/- mice, suggesting the potential effectiveness of this compound in constitutively activated systems.

In addition to inhibiting the active site of cGAS, another strategy is to disrupt the binding of cGAS to dsDNA, which is the trigger for cGAS activation. Antimalarial drugs (99, 100) such as hydroxychloroquine and quinacrine can inhibit IFNβ production by binding to dsDNA and preventing the formation of the cGAS-dsDNA complex. The specific mechanism of action is that these drugs intercalate between the base pairs of double-helical DNA, leading to helix unwinding (101). In addition, Wang et al. (102) found that suramin may inhibit cGAS activity by binding to the dsDNA binding site. The potential mechanism of action is that the anionic sulfate of suramin can act as a phosphate mimetic and bind to the positive region on cGAS. The authors also propose that the inhibition of cGAS by suramin is selective and has no effect on the TLR1, TLR2 or TLR4 pathways. Another cGAS antagonist is A151 (103), which is an inhibitory oligodeoxynucleotide containing 4 TTAGGG motif repeats (5′- tt agg gtt agg gtt agg gtt agg g-3′). It not only interacts with the dsDNA-binding domain but also inhibits the type I interferon response in TREX1-deficient cells, thereby increasing its potential to treat dsDNA-driven autoimmune diseases.

### 4.2 STING inhibitors

According to the available literature, there are two main types of STING inhibitors. The first approach is to design molecules that occupy the cyclic dinucleotide (CDN) binding site and become competitive antagonists of STING activators. The second approach is to find inhibitors that bind to Cys88 or Cys91 residues near the transmembrane regions of STING proteins, rendering them incapable of palmitoylation modification.

The major STING antagonists that target the CDN binding site are HSD1077 (104) and SN-011 (105). They compete with cGAMP for binding to STING and ultimately inhibit pro-inflammatory cytokine secretion. Small molecule STING inhibitor HSD1077, a novel quinoline-containing compound, is facilely synthesized through a Povarov–Doebner type three-component reaction involving an amine, ketone, and aldehyde. The compound is able to bind to STING and lead to a competitive displacement of cyclic dinucleotides. SN-011 is a potent and selective mouse and human STING inhibitor that binds the CDN-binding pocket with higher affinity than the endogenous cGAS product 2′3′-cGAMP and locks the STING dimer in an open, inactive conformation, thereby blocking expression of type I IFNs and proinflammatory cytokines in response to cytosolic DNA. SN-011 similarly inhibites activation of WT and SAVI-associated GOF mutants of STING.

Activation of the cGAS-STING pathway also requires palmitoylation of cysteine 88/91(Cys88/91) in the STING protein N-terminal transmembrane domain. Nitrofurans (C-176 and C-178) (106) specifically and irreversibly bind to Cys91 but not to Cys88 of STING. Pretreatment of mice with C-176 significantly reduced STING agonist-mediated increases of serum type I interferon and IL-6 levels. Compounds C-176 and C-178 are specific for mouse STING; however, the introduction of butyl and hexyl alkyl groups (C-170 and C-171, respectively) at the 4-position of the phenyl ring showed activity against both human and mouse STING. Similar to nitrofurans, the 3-acylamino indole derivative H-151 (106) also forms a covalent bond with Cys91, inhibits palmitoylation of STING Cys91, suppresses phosphorylation of TBK1, and reduces type I interferon responses. H-151 is active against both human and mouse STING. In addition, Vinogradova’s team found that the acrylamides BPK-21 and BPK-25 form adducts with STING’s Cys91 as well as with cysteines of other immune-related proteins (107). Hansen et al. reported that nitro fatty acids (NO2- FA), such as nitrooleic acid (NO2-OA) and nitrolinoleic acid (NO2- cLA), alkylate Cys88 and Cys91 in a non-selective manner. Their investigation further revealed that NO2-OA treatment nearly abolished the pTBK1 increase observed in fibroblasts from SAVI patients harboring the gain-of-function STING-N154S mutation (108).

### 4.3 STING agonists

STING agonists are developed for the treatment of malignant tumors, and there are several different viable strategies for targeting and modulating STING in the preclinical phase.

The most straightforward approach is the artificial synthesis of 2′,3′-cGAMP mimetics. ADU-S100 (ML RR-S2 CDA) is a synthetic CDN derivative with dithio mixed-linkages (109). Compared to endogenous CDNs, it has better stability and lipophilicity, significantly enhances STING signaling, and induces higher levels of IFN. It has been demonstrated that activation of STING with ADUS100 promotes the expression of IFN β and IL - 6, increases the number of CD8 + T cells and CD103 + dendritic cells, and inhibits the ex vivo and *in vivo* growth of cervical cancer cells (110). In addition, the replacement of two nucleosides (adenosine and guanosine) in natural CDNs with different sugar moieties (ribose, 2′-deoxyribose, or 2′-fluoro-2′-deoxyribose) yields a line of cyclic adenosine-inosine monophosphates (cAIMPs) analogs (111). Many of these cAIMP analogues significantly stimulate the IRF and NF-kB signaling pathways in human and murine immune cell lines and are more resistant to enzymatic cleavage *in vitro* than 2′,3′-cGAMP. Among these, cAIMPs 4 and 5, which contain two 2′-fluoro-2′-deoxyriboses, are the most potent in this series for inducing type I IFNs.

Furthermore, scientists have worked on the development of non-nucleotide small molecule STING agonists such as DMXAA (112), diABZI (113), SR-717 (114), and MSA-2 (115) to replace metabolically unstable nucleotide analogs. However, the affinity of non-nucleotide STING agonists for STING cannot be compared to that of naturally derived CDNs such as cGAMP. In a clinical trial in advanced non-small cell lung cancer, DMXAA administration failed to improve first-line antitumor efficacy due to its inability to bind with human STING (116, 117). The optimized diABZI showed 18-fold more potent than 2′,3′-cGAMP, but the membrane permeability remained a problem (118). Interdisciplinary collaborations including materials science, nanotechnology and pharmaceutics have become promising strategies to address the above challenges. Indeed, delivery systems for STING agonists, such as liposomal formulations and PLGA micro/nanoparticles, have been developed and used (119–121). Using nanoprecipitation methods, STING agonists were encapsulated in the hydrophobic core of micro/nanoparticles, thus effectively preventing their direct contacts or interactions with the physiological environment, and consequently, the bioactivity and half-life of STING agonists were highly improved. Furthermore, the micro/nanoscale delivery systems change the cellular uptake of STING agonists from direct diffusion to endocytosis, which improves the intracellular and presentation efficiency of antigen-presenting cells.

The combination of STING agonists and immune checkpoint inhibitors is gradually proving to be an effective treatment option against tumors. Tumor cells prevent the immune system from attacking them by expressing a large number of immune checkpoint molecules, such as PD-1, PD-L1, CTLA-4, and so on. The application of immune checkpoint inhibitors can relieve the inhibition of the immune response by the tumor and enable the immune response induced by the cGAS-STING pathway. Similarly, activation of STING can drive a strong anti-tumor immune response and increase tumor sensitivity to immune checkpoint inhibitors (122–124). The effectiveness of this combination has been further confirmed by some recent studies. A mutant type of HPV 16 E7 protein (E7GRG) combined with the two 2′~ 3′ cGAMP and CpG - C adjuvants could induce strong cellular and humoral immune responses and mediate tumor growth suppression in mouse models. This vaccine formulation may be a promising therapeutic candidate vaccine for HPV 16 established infections and HPV-associated tumors (125). The immune effects of chemotherapeutic drugs such as cisplatin and etoposide may be partially related to the cGAS/STING signaling pathway. These drugs cause DNA leakage into the cytosol, intrinsically triggering cGAS/STING signaling (68, 126). It has also been shown that cGAS/STING signaling is essential for effective radiation therapy. DNA that accumulates in the cytosol upon radiation stimulates interferon secretion by cancer cells following activation of the DNA sensor cGAS and its downstream effector STING. Repeated irradiation amplifies interferon production, leading to the recruitment and activation of Batf3-dependent dendritic cells (6, 72, 127, 128). The latest research also indicates that activation of STING-TBK1 (TANK-binding kinase 1) promotes the ubiquitin-proteasome degradation of the E7 oncoprotein, thereby inhibiting the growth of cervical cancer. Mechanistically, TBK1 can phosphorylate the HPV16/18 E7 oncoprotein at Ser71/Ser78 sites, facilitating the ubiquitination and degradation of E7 by the E3 ligase HUWE1. Functionally, activated STING inhibits cervical cancer cell proliferation by downregulating E7 oncoprotein in a TBK1-dependent manner and potentially synergizes with radiation to achieve better effects for antitumor (129).

## 5 Conclusions and perspective

The cGAS-STING axis plays a crucial role in protecting higher organisms from invading pathogen or cancer by promoting the production of cytokines and interferons. Its significance is particularly evident in female reproductive system diseases, where the cGAS-STING pathway is vital for maintaining normal reproductive function and immune homeostasis. Although the activation of cGAS-STING has its benefits, abnormal activation and dysfunction of this axis lead to chronic upregulation of cytokine expression, which has been shown to play a key role in the development of chronic autoimmune diseases. In conditions like endometriosis and adenomyosis, excessive activation of the cGAS-STING pathway has been observed, leading to enhanced inflammation and immune responses, which promote abnormal growth of lesions. The discovery of this mechanism provides new insights for treating these diseases with unclear etiology and complex histology, and the cGAS-STING pathway is viewed as an attractive target to ameliorate the symptoms associated with these autoimmune disorders.

Conversely, in ovarian, cervical, and endometrial cancers, the activity of the cGAS-STING pathway is suppressed, hindering the expression of tumor-associated antigens and the activation of immune cells, which allows cancer cells to evade immune system attacks. A promising approach to improving cancer immunotherapy is enhancing the innate immune system’s ability to recognize and mount an immune attack against cancer cells through immune adjuvants. Cytokines secreted after cGAS-STING pathway activation can activate immune cells such as T cells and NK cells to identify and eliminate cancer cells. Due to the strong immunostimulatory effects of the STING pathway, its agonists have become promising drugs in the field of cancer immunotherapy. Additionally, because of its physiological function in innate immunity, STING agonists are often used in combination with immune checkpoint inhibitors (ICIs). Accumulated clinical evidence has confirmed that the combination of ICIs and STING agonists is key to enhancing immune responses and improving the effectiveness of cancer immunotherapy. In summary, elucidating the potential mechanisms of the cGAS-STING pathway under pathological conditions will help regulate immune imbalance and inflammatory responses in patients, thereby preventing or treating female reproductive system diseases. This gives us a new breakthrough point in managing these diseases more effectively.

However, the role of cGAS/STING-mediated signaling in diseased cells still remains largely unknown at present. The source of dsDNA that activates cGAS and the downstream signaling pathways that promote disease development have not been comprehensively elucidated. Moderate activation of the cGAS-STING pathway generates immune defense, but excessive activation triggers inflammatory responses. How to control the activation degree of the cGAS-STING pathway and whether there are negative regulatory mechanisms to limit its overactivation remain unknown. Researchers can explore possible negative regulators and regulatory signaling pathways as well as the impact of the imbalance in the regulation of the cGAS-STING pathway on the body’s immune balance.

In addition, it is not known if the downregulation of cGAS/STING expression and impaired signaling in cancer cells creates a survival advantage or if it is just a non-important by-product of carcinogenesis. The functional consequences of cGAS/STING activation in cancer cells are also unknown. It needs to be verified whether the induction of STING-mediated signaling in cancer cells could lead directly to cancer cell death or merely to cytoprotection.

Differences in the regulatory mechanisms of the cGAS-STING pathway between different tumor types should also be explored. Just as the efficacy of immune checkpoint inhibitors varies in different types of tumors, so does the extent of immune cell activation by the cGAS-STING pathway. This may be due to the distinct immune microenvironment in different types of tumor cells, causing differences in the regulatory mechanisms of the cGAS-STING pathway. Therefore, studying the discrepancy in the role of the cGAS-STING pathway in different types of tumors can help to understand the mechanisms of tumor immune escape and design more individualized therapeutic regimens.

Finally, it will be important to note that despite the potential of the cGAS-STING pathway in the treatment of female reproductive system diseases, specific applications are currently in the exploratory phase. Further studies are needed to determine the optimal therapeutic strategies and to assess their safety and tolerability in patients. Advances in these researches will provide the theoretical basis for related disease treatment options.
